# Automated method to differentiate between native and mirror protein models obtained from contact maps

**DOI:** 10.1371/journal.pone.0196993

**Published:** 2018-05-22

**Authors:** Monika Kurczynska, Malgorzata Kotulska

**Affiliations:** Department of Biomedical Engineering, Faculty of Fundamental Problems of Technology, Wroclaw University of Science and Technology, Wroclaw, Poland; UMR-S1134, INSERM, Université Paris Diderot, INTS, FRANCE

## Abstract

Mirror protein structures are often considered as artifacts in modeling protein structures. However, they may soon become a new branch of biochemistry. Moreover, methods of protein structure reconstruction, based on their residue-residue contact maps, need methodology to differentiate between models of native and mirror orientation, especially regarding the reconstructed backbones. We analyzed 130 500 structural protein models obtained from contact maps of 1 305 SCOP domains belonging to all 7 structural classes. On average, the same numbers of native and mirror models were obtained among 100 models generated for each domain. Since their structural features are often not sufficient for differentiating between the two types of model orientations, we proposed to apply various energy terms (*ETs*) from PyRosetta to separate native and mirror models. To automate the procedure for differentiating these models, the k-means clustering algorithm was applied. Using *total* energy did not allow to obtain appropriate clusters–the accuracy of the clustering for class A (all helices) was no more than 0.52. Therefore, we tested a series of different k-means clusterings based on various combinations of *ETs*. Finally, applying two most differentiating *ETs* for each class allowed to obtain satisfying results. To unify the method for differentiating between native and mirror models, independent of their structural class, the two best *ETs* for each class were considered. Finally, the k-means clustering algorithm used three common *ETs*: probability of amino acid assuming certain values of dihedral angles *Φ* and *Ψ*, Ramachandran preferences and Coulomb interactions. The accuracies of clustering with these ETs were in the range between 0.68 and 0.76, with sensitivity and selectivity in the range between 0.68 and 0.87, depending on the structural class. The method can be applied to all fully-automated tools for protein structure reconstruction based on contact maps, especially those analyzing big sets of models.

## Introduction

Mirror-image proteins may soon become a mile stone in biochemistry. Mirror reflection of a native protein may function in the same way as the native protein, however they may be resistant to viruses and molecules which are not compatible with mirror-image structures. The first step in the mirror-image biochemistry has been already made. Mirror polymerase, which copies left-handed DNA, was created [1]. Also molecular dynamics studies showed that mirror proteins may be competitive forms in nature, due to their thermodynamic stability [2,3,4]. Moreover, some types of proteins may have the same properties in both orientations. For example, antimicrobial peptides, which may be next-generation therapeutics for drug-resistant bacteria [5], have similar binding affinity to the membrane in both forms, independent of their chirality [6].

The mirror aspect of a protein may relate to an ideal reflection of a native protein which could be built from D-amino acids instead of L-amino acids. It may also concern a mirror arrangement of the domains (tertiary structure), as in the study by Noel et al. [2], or a secondary structure of a protein, for example reversed handedness of a helix. The last case is well known from modeling unknown protein structures from contact maps.

Protein structures whose backbones are mirror images of each other generate identical contact maps between Cα or C_β_ atoms in the protein backbone [7]. Notably, both orientations may be chemically stable although only one exists in the nature. It poses a problem for methods using contact maps for protein modeling. Computational methods for protein structure modeling based on contact maps usually generate a set of tentative models which belong to both orientations. It is not always obvious which models should be filtered out with regard to their orientation, especially if the procedure is supposed to be fully automatic and applicable to large sets of models.

Development of the methods based on contact maps improves modeling of unknown protein structures but it still has not brought satisfying approach capable of differentiating between native and mirror models [8–12]. Some methods of model generation use chirality-related terms, which help to avoid left-handed helices [13,14]. However, these methods are not useful for proteins rich in beta-sheets. Another idea was based on torsion angles and modifying a structure according to the allowed values of the torsion angle [15]. Another group of methods compare models to their native structures, using root mean square deviation (RMSD), clustering [16], or ranking models [9]. These methods, however, require the knowledge of the native protein structure. Moreover, this approach can influence the assessment of the reconstruction method, because it rejects the worst models, but not necessarily mirror models. Therefore, another method differentiating between both types of model orientations is needed. In our previous preliminary study [17] we showed that energy terms (*ETs*) from PyRosetta could be suitable to address this problem for selected proteins rich in alpha-helices. However, proteins rich in beta-sheets are harder cases for straightforward differentiation between native and mirror models. The GDFuzz3D tool [12] proposed to use a scoring function of different tools to choose the appropriate chirality of the model. However, Pietal et al. [12] mentioned about a mismatch between global and local handedness of models which may deteriorate the effect.

In this study we made a thorough systematic analysis of protein structures—representatives of all different structural classes, including alpha-helices and beta-sheets. We used 1 305 SCOP protein domains, each represented by 100 models. The models were obtained using knowledge-based potentials deduced from known protein structures. As this is a usual case in this kind of modeling, the result includes also protein models with backbones of the mirror orientation although other parts of the proteins are oriented correctly.

Our main goal was to propose an automated method able to differentiate between native and mirror models, based on their energy terms *ETs*, and independent of the class of their secondary structures.

## Results and discussion

### Structural characteristic of mirror models in relation to native models

**Fig 1:**
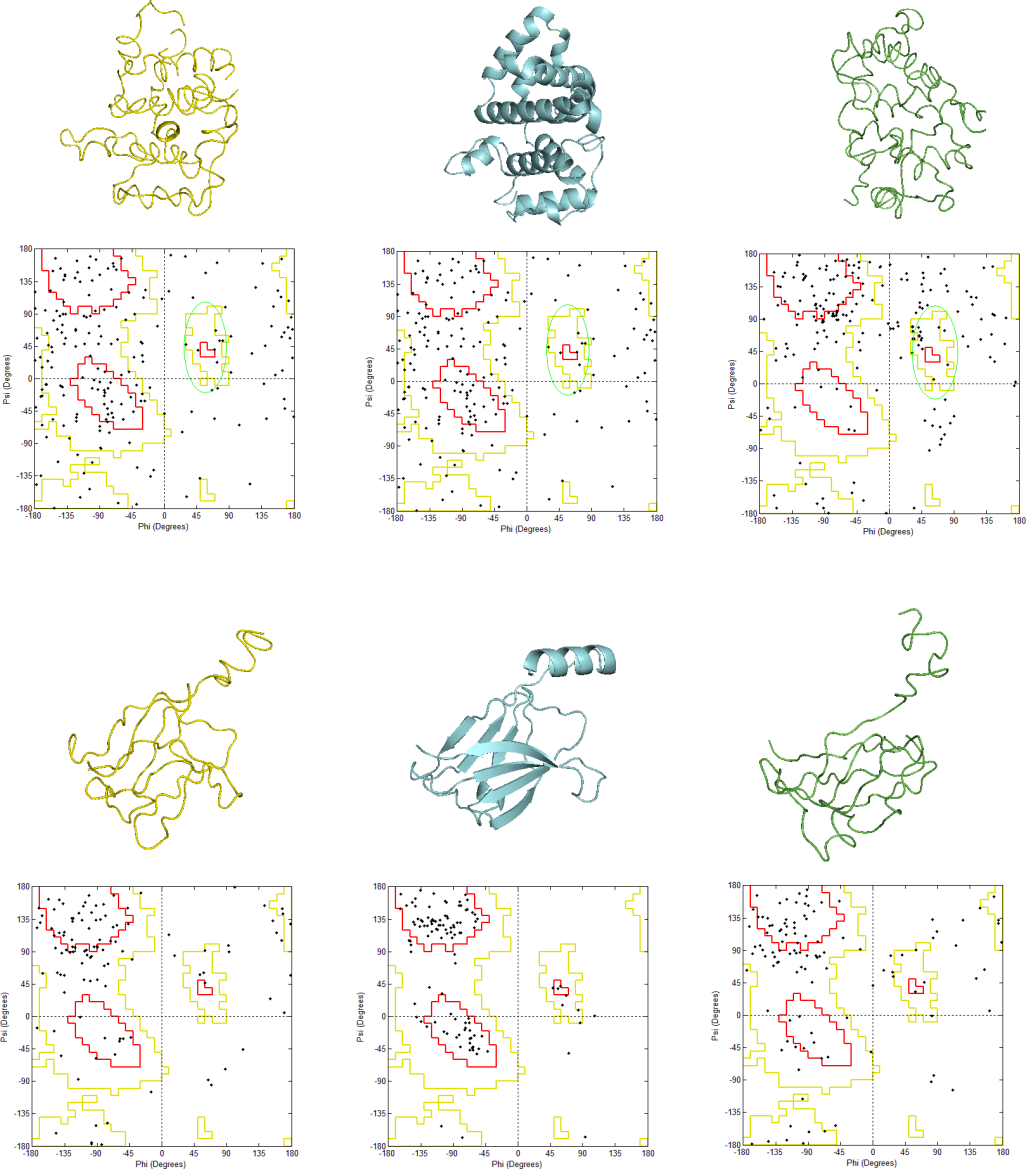
**Exemplary models of the domains: a) A class d1tx4a_, b) B class d1osya_.** The yellow structures are natively oriented models (left), the blue structures are SCOP structures (middle), and the green structures are mirror models (right). Their Ramachandran plots are presented below the structures. Red area is the general favored region, yellow area is the allowed region, and black points denote residues.

The problem of generating a variety of models from their contact maps is illustrated in Fig 1. A lot of tools for protein reconstruction from contact maps use known structures from databases. This solution is less time-consuming than classical *de novo* modelling. The Cα-traces obtained from such modeling may have a native or a mirror form, but the rest of the protein structure is usually built from rotamers or different fragments of the known proteins. Therefore, the mirror models regarded here are not ideal reflections–the mirror orientation concerns only a secondary structure (more details about our protein structure reconstruction from contact maps in Materials and Methods).

Two protein domains, presented in the middle of the picture (blue structures), were modeled. Since contact maps are identical for two chiral forms of a protein, we obtained native models (left, yellow structures) and mirror models (right, green structures). The domain from class A (Fig 1A) is rich in alpha-helices, which is also seen in the Ramachandran plot. The original SCOP structure and the model with the native orientation have right-handed helices, while the mirror model has left-handed helices. Based on the criteria described in Methods, 97% of residues of the original SCOP structure locate in the favored region, 1.0% in allowed and 2.0% in the outliers region. Most of the residues are placed in right-handed helices. A majority of residues of the natively oriented model locate nearby the region of the right-handed helices in the Ramachandran plot, which means allowed and favored regions. Conversely, the residues of the mirror model place in different regions of the Ramachandran plot. Many residues from the region of negative dihedral angles (3^rd^ quarter) are moved to the region of positive dihedral angles (1^st^ quarter). As a result more residues locate in the region of left-handed helices, which is marked by the green ellipse. The structure from class B (Fig 1B), as well as its models, are similar to each other while assessed visually. We did not observe a significant transition of the residues from the 3^rd^ quarter to the 1^st^ quarter in the Ramachandran plots as it was the case for the models rich in alpha-helices. Moreover, both models of the beta-sheets had similar numbers of the residues in outliers’ regions. These results suggest that structural features are not sufficient for selection of the mirror models of all types of structures.

In the analyses we used protein models, so we verified differences in contact maps of finally generated and refined models in relation to their original SCOP structures. We observed that the models from class B preserved more contacts of their original SCOP domains than class A (S1 Table). The median differences (*diff*) between maps of models and their original SCOP domains was 0.4% for class B and 0.9% for class A. 16% of models in class B maintained their original contact maps, while only 5% in class A. This shows that models with beta-sheets were better fitted to the original contact maps than the models with alpha-helices. We did not observe any significant differences between contact maps of the native and mirror models.

For comparative analysis we needed to know if the ratio of mirror models was constant. Using C2Sv2.0 we obtained that both orientations of models were equally likely. Mirror models constituted ca. 50% of all models, when median was considered. However, in each SCOP class we observed outliers. There were domains whose ratio of mirror models was close to 0 or 1. Only 2.5% of domains had only one type of model orientation: 1% of domains did not have any mirror models and 1.5% of domains had only mirror models. The distribution of the ratio of the mirror models for all domains was Gaussian, which was supported by the Jarque-Bera test. There were no correlations between the ratio of mirror models and other features of the domains, such as a domain length (number of residues), a difficulty in modeling the structure (RMSD of native models to the original SCOP structure), and a structural difference between the original SCOP structure and its mirror image (RMSD of the original SCOP structure to its mirror).

**Fig 2:**
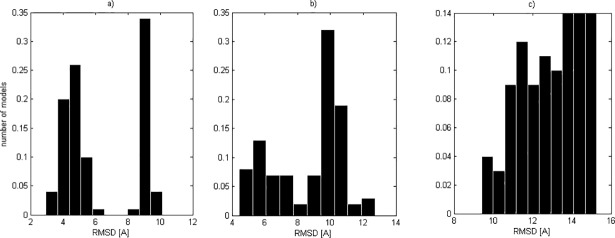
Histograms of RMSD models to the SCOP structures demonstrating structural differences between native and mirror models: a) different (domain d1hx1b_), b) similar (domain d1boua_) and c) very similar (domain d1a9xa1).

In our study we worked with 130 500 models, so visual assessment of each model would need a huge amount of time. For this reason we used RMSD of models to compare the structures. The histogram of RMSDs of each domain may demonstrate structural differences between both types of model orientations. However, three histogram types were observed (Fig 2). The first histogram (Fig 2A) shows the domain whose native and mirror models were the most distinct. The shape of the RMSD histogram hints at two separate distributions. The second type of RMSD histogram has two overlapping distributions (Fig 2B). The native and mirror models may be still separated. However, some models are in the ‘gray area’ and it is hard to assess if they are natively oriented or mirror. The third histogram (Fig 2C) shows the most indistinguishable models. The single unimodal RMSD histogram does not indicate which models are natively oriented and which are mirror. Moreover, the more difficult was the structure for modeling, the more alike were RMSDs of natively oriented and mirror models.

The mean RMSD of models was calculated for each domain. The mean value of domains for each class is shown in Table 1. The mean RMSD of all models compared to their original SCOP structures was 8.9 Å ± 5.3 Å. The lowest mean RMSD value was observed for G class, which was related to the shortest sequences of the domains. The highest mean RMSD values were recognized for E and F classes. E class included the longest sequences of the domains. However, the high mean RMSD and the highest differentiation in qualities of the models for F class, which contained the domains of membrane and cell surface proteins and peptides, suggested that some of the domains were difficult to reconstruct.

**Table 1:** The mean RMSD of models of each class.

SCOP class	All models to SCOP	Mirror models to SCOP	Native models to SCOP	Mirror models to mirror SCOP
A	9.4 ± 5.0	11.9 ± 4.7	6.8 ± 5.4	6.8 ± 5.4
B	8.5 ± 4.5	12.7 ± 4.1	4.4 ± 3.8	4.5 ± 4.4
C	9.5 ± 3.6	14.7 ± 2.4	4.1 ± 2.4	4.1 ± 2.4
D	8.4 ± 3.7	12.1 ± 3.1	5.0 ± 3.6	5.0 ± 3.6
E	11.5 ± 5.5	16.8 ± 3.3	7.0 ± 4.7	7.6 ± 5.0
F	12.1 ± 12.3	13.7 ± 12.2	10.3 ± 12.6	10.2 ± 12.4
G	6.6 ± 5.3	8.7 ± 5.3	4.7 ± 5.4	4.7 ± 5.8
**All**	**8.9 ± 5.3**	**12.3 ± 5.1**	**5.6 ± 5.4**	**5.6 ± 5.5**

Assessing quality of models by comparing the mirror models to their original SCOP structures may be misleading. Dividing the set of the models into two groups of model orientations, the mean values of RMSD was lower for natively oriented models (5.6 Å ± 5.4 Å) than for mirror models (12.3 Å ± 5.1 Å). Therefore, the mirror models should be assessed separately in relation to the mirror image of the original SCOP structure. The mean RMSD of the mirror models to the SCOP mirror was the same as the mean RMSD of the natively oriented models to the original SCOP structure (5.6 Å). Additionally, almost in each class the mean RMSD values of both model types were the same (S1 Fig).

Therefore, we confirmed theoretical considerations about random chirality of models obtained from contact maps presented in [18]. The results showed that 50% of models are usually natively oriented and, moreover, the quality of their structural features is the same as that of the mirror models. However, the number of natively oriented models of some proteins may be different than 50%. Therefore the post-generating procedure applied by Darute et al. [9], which always retains one-third of the models with the lowest RMSD as natively oriented models, may give a bias in the final results.

### Ramachandran plots for natively oriented and mirror models

**Fig 3:**
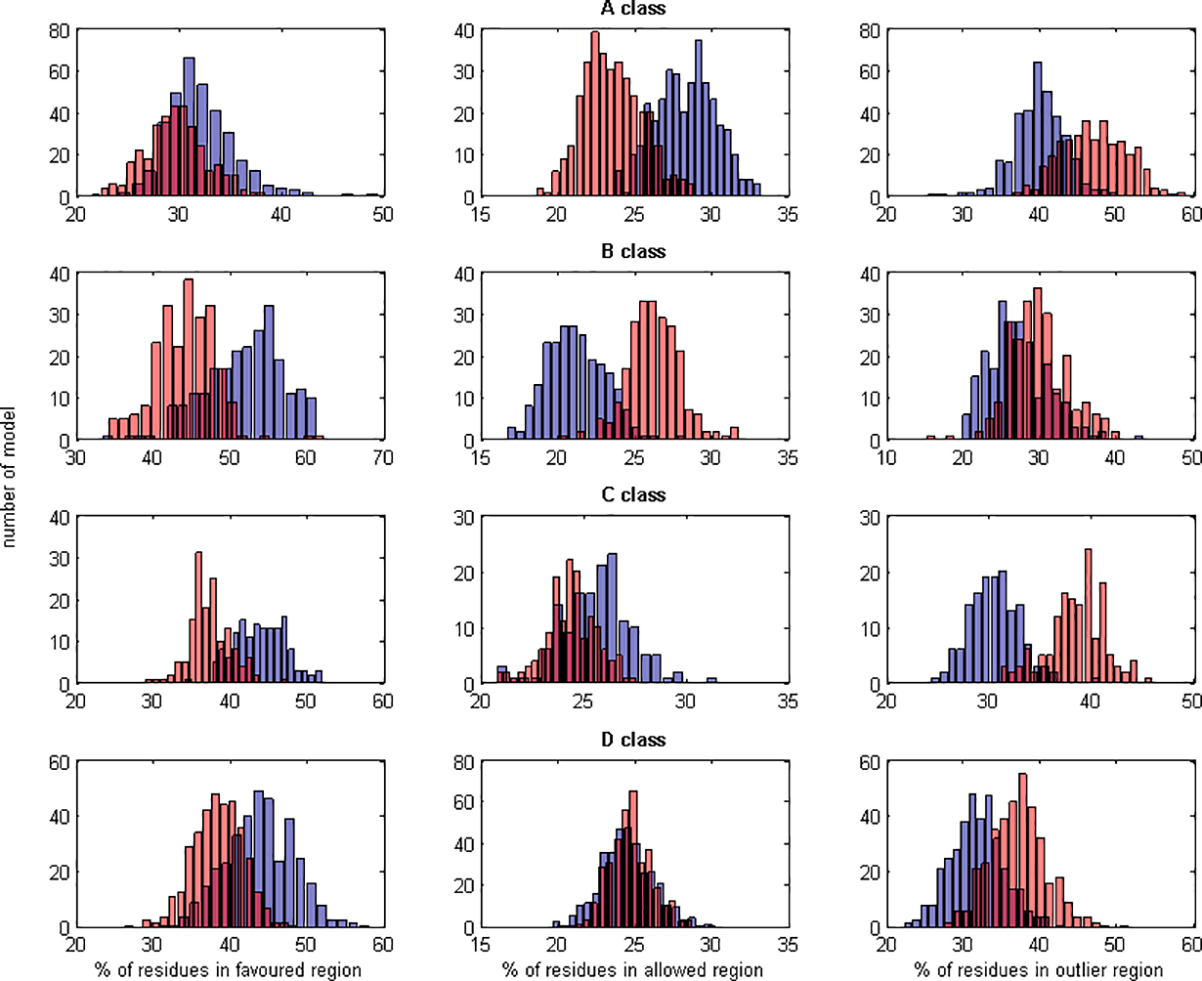
**Mean differences in Ramachandran plots between natively oriented (blue bars) and mirror models (red bars) of domains in A, B, C and D classes.** Histograms of mean percentages of residues in favored (left), allowed (middle) and outlier (right) regions.

In the analysis of differences between natively oriented and mirror models, the Ramachandran plots were considered. Residue coverages of the favored, allowed and outlier regions were calculated for each model and mean values for each domain were calculated. The histograms of the residue coverage are presented in Fig 3 (only A, B, C and D classes are shown).

The mean residue coverage of the allowed region, for all models, was in the range from 21.1% to 28.3%. Four classes (C, D, E, and G) had similar proportions between the mean residue coverage of the favored (ca. 40%) and outlier regions (30%). However, this proportion was different for three other classes (A, B and F). Class A, which included all-alpha domains, and class F which included membrane and cell surface proteins had more residues in the outlier regions (ca. 40%), and fewer in the favored regions (ca. 30%). Conversely, the mean residue coverage in class B was the highest for the favored region (51.6%). Only 26.9% of residues in class B were in the outlier region.

The Ramachandran plot analysis may answer the question if the distributions of the residue coverages are different for natively oriented and mirror models (red and blue bars in Fig 3). We observed that there was no uniform rule for all classes. The residue coverages of the favored region were similar for natively oriented and mirror models in class A. The reason for this was the fact that the left-handed helices are also deposed in the databases. However, in case of B, C and D classes they were higher for natively oriented models than for mirror models. In the allowed region the residue coverage was higher for mirror models in class B, while this relation was opposite for class A. For C and D classes the differences were minor. On the other hand, the outlier regions of all classes were dominated by the mirror models.

Due to the diversity of Ramachandran plots for different classes their use for discriminating between natively oriented and mirror models is limited. Therefore, the methods based on adjustment of torsion angles to specific values [15,16] are insufficient.

### PyRosetta energy terms as an indicator of mirror models

Since structural features are not sufficient to distinguish between natively oriented and mirror models, we applied *ETs* from PyRosetta to this problem. The *total* energy of a protein model did not indicate the model orientation in each case. We observed a wide range of percentages of domains for which the *total* energy was differentiating between two model orientations, according to the structural class of the protein. In classes A and F the *total* energy was statistically different between natively oriented and mirror models only for 37% and 22% of domains, respectively. On the other hand, in classes B, C and D the *total* energy was differentiating for more than 75% of domains. For that reason we studied each *ET* in each structural class of proteins to test if it is significantly different for natively oriented and mirror models.

**Fig 4:**
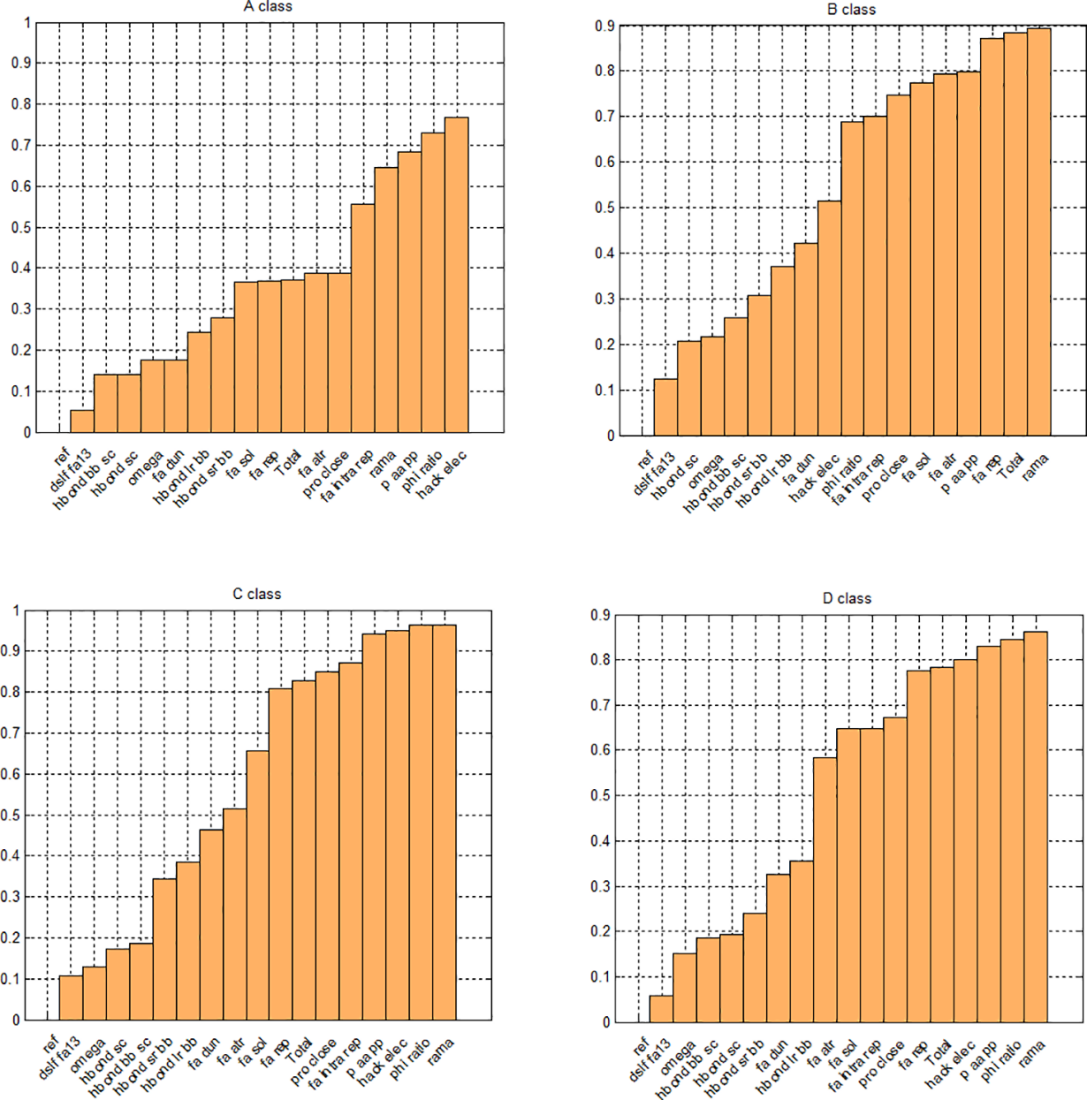
Bar graph showing the ratio of domains for which the ET was significantly different in the groups of natively oriented and mirror models. Graph includes also *Φ*^*+*^ *ratio*.

In the set of *ETs* several energy terms were significantly different for more than 60% of domains in each structural class. We observed that some empirical potentials (e.g. *hack_elec*, *fa_rep*) were differentiating for both types of the model orientations, similarly to some knowledge-based potentials (e.g. *rama*, *p_aa_pp*). Fig 4 shows the ratio of domains for which the *ETs* were significantly different between natively oriented and mirror models. Fig 4 includes domains from classes A, B, C and D. The results for the domains from classes E, F and G are presented in Supplementary Materials (S2 Fig).

For domains from class A the most differentiating terms were *ETs* describing electrostatic interactions (*hack_elec*), which were different for more than 77% of domains, and dihedral angles (*p_aa_pp*– 68%, and *rama*– 64%). Notably, in class B the number of the *ETs* which were differentiating for more than 60% of domains was twice as high as in class A. Moreover, the ratio of the domains for which the *ETs* were significantly different between natively oriented and mirror models was higher in class B than in class A. More than 89% of domains in class B had significant differences in Ramachandran preferences (*rama*). The differences between classes A and B for *rama* may be an effect of the structures deposited in databases, where we can also find left-handed alpha-helices. Another *ET* related to the dihedral angles (*p_aa_pp*) was also useful for more than 80% of domains. Furthermore, the Lennard-Jones repulsive (87%) and attractive (79%) terms were significantly different for domains from class B. Conversely than in class A, we did not notice significant changes of electrostatic interactions (*hack_eleck*) for mirror models in relation to the natively oriented models. Three structural classes C, D and E included alpha-helices and beta-sheets, so for them we noted similar usability of the same *ETs* as in class B, including also electrostatic interactions typical of class A. Classes F and G were the hardest to find their differentiating *ETs*. We did not obtain any *ET* which was different for more than 60% of domains in class F and we found that only *rama* was different for 60% of domains in class G.

**Fig 5:**
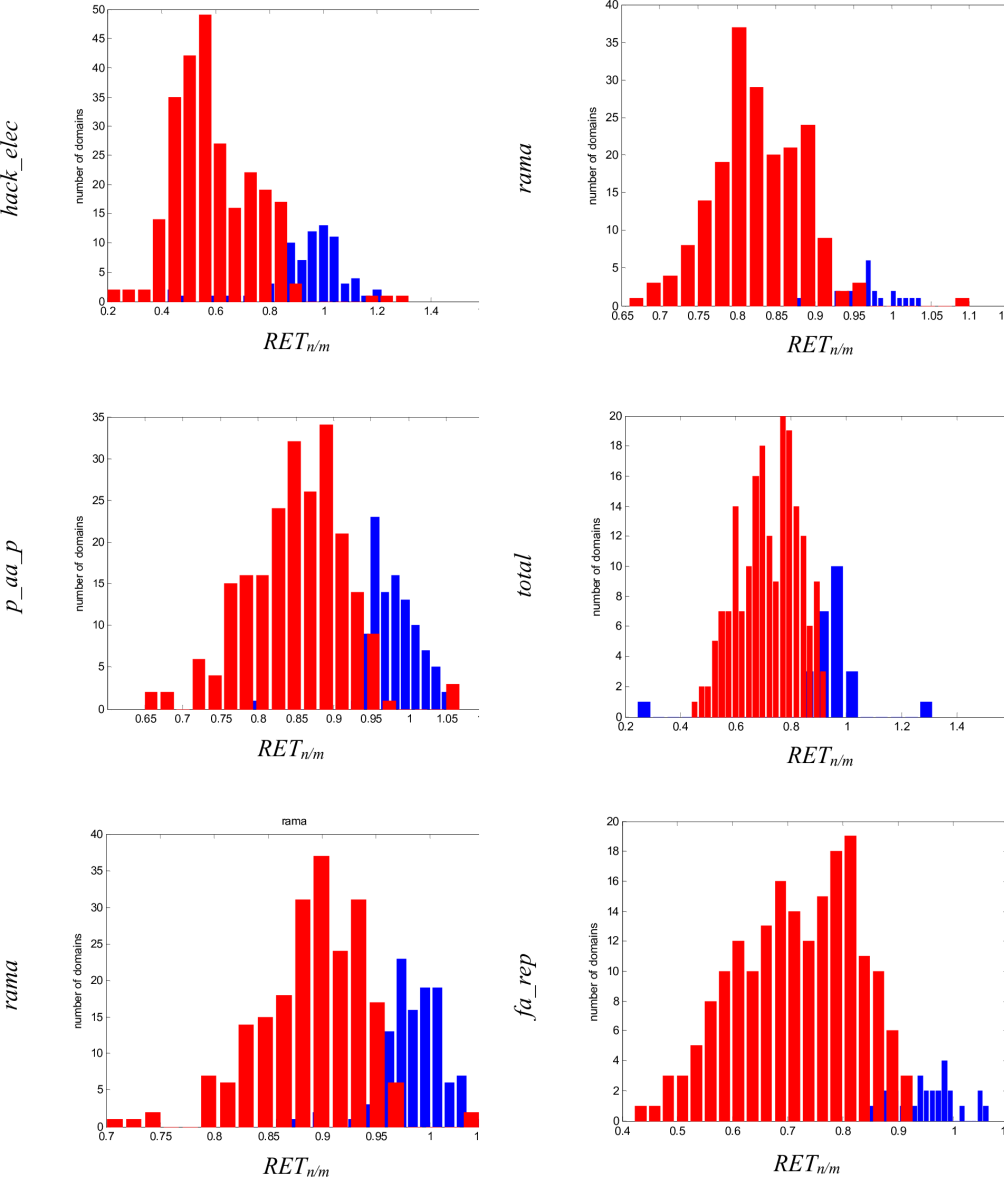
The histograms of RET for the three best ET in distinguishing native and mirror models. Left column is class A and right column is class B, red bars mean the domain for which the ET was significantly different and blue bars mean the domain for which the ET was not significantly different.

We tested if natively oriented models had lower values of these *ETs* which were significantly different. The histogram of the relation between energy terms *RETs* (see Eq 4 in Materials and Methods) of the most reliable *ETs* for class A and B is shown in Fig 5. The *RETs* histograms of the rest of classes are shown in the Supplementary Material (S3–S5 Figs). The domains for which *ETs* were statistically different for native and mirror models are colored in red. The domains for which *ETs* were not statistically different for both types of model orientations are colored blue. The majority of domains from class A and B had lower values of *ETs* for natively oriented models, which means that they were more stable (*RET* lower than 1). Only a few domains had higher values of *ETs* for natively oriented models, and most of them were colored blue, for them there were no significant differences between natively oriented and mirror models.

**Fig 6:**
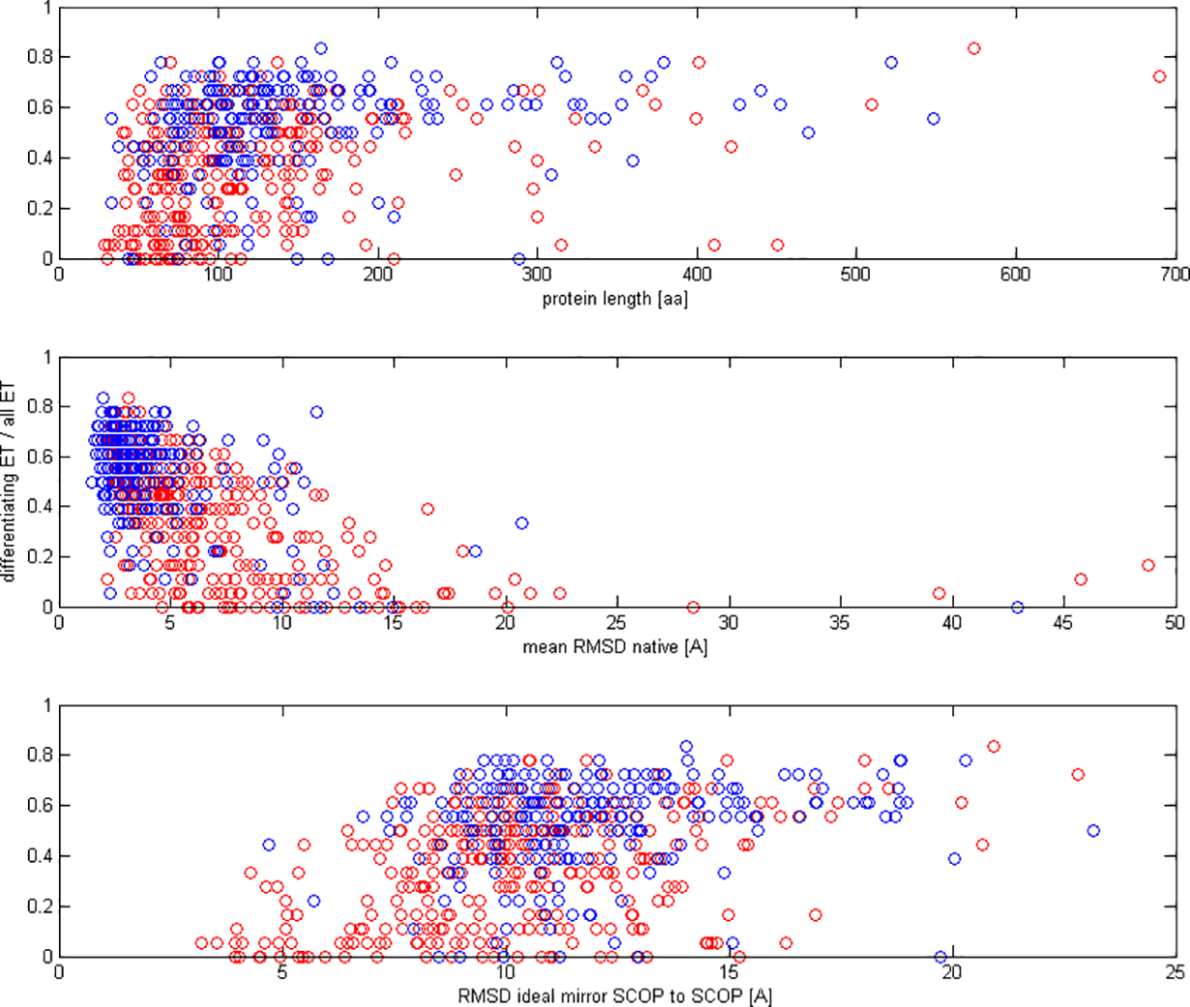
Scatter plot of differentiating ETs to all ETs vs. protein length, mean RMSD of native models (mean by domains) and RMSD between SCOP structure and its mirror: Red is class A, blue is class B.

We studied if the number of the *ETs* which were significantly different for natively oriented and mirror models was dependent on the structural features of proteins (Fig 6). We observed a week correlation (*τ* = 0.30) between the number of differentiating *ETs* and the length of the protein sequence. If a protein was more difficult in modeling, expressed by RMSD of natively oriented models to the original SCOP structure, fewer *ETs* were differentiating between native and mirror models (*τ* = −0.40). Nonetheless, the correlation was weaker for class B (*τ* = −0.23) than for class A (*τ* = −0.47). We also observed a weak correlation between the number of differentiating *ETs* and RMSD of the original SCOP structure to its mirror. More *ETs* were differentiating when the RMSD had a greater value (*τ* = 0.28).

In our study we also used another method for differentiating two types of model orientations in order to validate the approach based on ETs. We applied the unsupervised k-means clustering algorithm, based on the differentiating *ETs*, in order to classify the data set into mirror and native models. The mean accuracies, specificities, sensitivities, F1 and MCC of clustering for all classes are shown in Table 2.

**Table 2:** Mean accuracy of the k-means clustering using energy terms.

SCOP class		A	B	C	D
All *ETs*	*ACC*	0.53	0.75	0.67	0.68
	*SPC*	0.50	0.74	0.67	0.67
	*SN*	0.59	0.81	0.74	0.73
	*MCC*	0.08	0.51	0.36	0.36
	*F1*	0.53	0.75	0.65	0.68
*ETs* different for > 60% domains	*ACC*	0.68	0.76	0.67	0.68
	*SPC*	0.72	0.74	0.67	0.67
	*SN*	0.69	0.82	0.74	0.74
	*MCC*	0.38	0.52	0.36	0.37
	*F1*	0.66	0.75	0.65	0.69
2 most differentiating *ETs*	*ACC*	0.71	0.75	0.78	0.68
	*SPC*	0.73	0.75	0.87	0.76
	*SN*	0.72	0.81	0.77	0.66
	*MCC*	0.43	0.51	0.59	0.38
	*F1*	0.69	0.75	0.75	0.66
1 most differentiating *ETs*	*ACC*	0.71	0.72	0.72	0.68
	*SPC*	0.69	0.81	0.84	0.76
	*SN*	0.77	0.69	0.70	0.66
	*MCC*	0.43	0.45	0.50	0.39
	*F1*	0.70	0.69	0.68	0.66
1 *ET*: *total*	*ACC*	0.52	0.76	0.67	0.68
	*SPC*	0.47	0.75	0.67	0.67
	*SN*	0.59	0.82	0.74	0.73
	*MCC*	0.05	0.52	0.36	0.36
	*F1*	0.52	0.75	0.65	0.68
3 *ETs*: *hack_elec*, *p_aa_p*, *rama*	*ACC*	0.68	0.70	0.76	0.70
	*SPC*	0.72	0.77	0.87	0.78
	*SN*	0.69	0.69	0.74	0.68
	*MCC*	0.38	0.42	0.56	0.42
	*F1*	0.66	0.68	0.72	0.68

When all *ETs* were involved, the highest accuracy was noted for class B (0.75) and the lowest accuracy for class A (0.53). The accuracies for clustering in classes C and D were between the results of classes A and B. Then, we limited *ETs* to these which were significantly different for more than 60% of domains. The accuracy of clustering was better for class A (0.68), but the accuracies for different classes did not change. Next, we used only two most differentiating *ETs*. The results were higher for classes A and C. However, the accuracies for B and D classes were the same as in the case with all *ETs*. Therefore, using only two most differentiating *ETs* in k-means clustering for each class allowed us to obtain two groups of models with accuracy between 0.68–0.78, specificity 0.73–0.87, and sensitivity 0.66–0.81, depending on the class. However, we did not find one common *ET* for all classes (*ETs* for class A: *hack_elecl* and *p_aa_p*, for class B: *rama* and *total*, for class C: *rama* and *hack_elec* and for class D: *rama* and *p_aa_p*).

We addressed a question if using only *total* energy would allow obtaining appropriate clusters. The accuracy of the clustering for class A was only 0.52, while the accuracies of clustering models in classes B and D were more acceptable (0.67–0.76). Next, we evaluated if clustering based only on one ET (best for each class) would be sufficient for distinguishing between natively oriented and mirror models. For class A we used *hack_elec*, for class B, C and D *rama*. The results for classes A and D were the same as in the case with two most differentiating *ETs*. However, the accuracies of clusterings for classes B and C were slightly better in the case of two *ETs*.

Our idea was to find common *ETs* for all structural classes of proteins. For that reason out of all *ETs* which were used in clustering, two most differentiating *ETs* for classes A, B, C and D were selected. Despite the fact that *total* energy was one of two most differentiating *ETs* for class B, we excluded it, because of the low accuracy in class A. As a result we obtained three common *ETs* for all classes: *hack_elec*, *p_aa_p*, and *rama*. The mean accuracy of clustering with three *ETs* for classes A, B, C and D was 0.71 (details in Table 2). Clustering with two most differentiating *ETs* for each class and clustering with three common *ETs* gave similar results. Therefore, using the same *ETs* for all domains allow to simplify the automated method for differentiating natively oriented and mirror models.

The clustering results for classes E, F and G are included in Supplementary Materials (S2 Table).

The models were divided into 2 classes. Only domains with at least 3 mirror and 3 native models were included. Squared Euclidean distance was used. ACC denotes accuracy (Eq 5), SPC denotes specificity (Eq 6), SN denotes sensitivity (Eq 7), MCC denotes Matthews correlation coefficient (Eq 8), and F1 denotes F1 score (Eq 9).

## Conclusions

The visual assessment of the structures rich in alpha-helices and their Ramachandran plot may give a clue which model has the native orientation of its secondary structure and which is closer to the mirror image. However, the a priori knowledge about chirality of the original helices is necessary. Proteins rich in beta-sheets are harder cases for visual differentiation between native and mirror models. Moreover, total energies of the structures are not always helpful. Therefore, we proposed the automated method for differentiating both types of model orientations independent of their secondary structures.

We analyzed protein models of a set of SCOP domains from seven structural classes. On average the same numbers of natively oriented and mirror models were obtained, and the distribution of the ratio of mirror models to all models was Gaussian. This confirms the assumption about the same probability of both types of model orientations proposed in [18], when no extra chirality terms are used in the tool for protein structure reconstruction from contact maps. We showed that the structures rich in beta-sheets preserved more original contacts during reconstruction process than those with predominant alpha-helices. To assess the models, their RMSDs to the original SCOP structures were used. We observed three types of RMSD histograms of the models: separated bimodal distribution, overlapping bimodal distribution, and unimodal distribution. All of them present three levels of difficulty in structural differentiation between natively oriented and mirror models.

Since the structural features are not sufficient for differentiating between both types of model orientations, we proposed to apply energy terms from PyRosetta. The *total* energy was statistically different between natively oriented and mirror models only for 37% of domains rich in alpha-helices, but for 89% of domains rich in beta-sheets. Therefore, we analyzed each *ET* separately. For domains from class A the most differentiating terms were: *hack_elec*, *p_aa_p* and *rama*, which describe Coulomb interaction, probability of amino acid at defined values of dihedral angles and Ramachandran preferences. For domains from class B they were: *rama*, *p_aa_p*, such as in class A, and *fa_rep*, *fa_atr*, which describe Lennard-Jones repulsive and attractive interactions.

We applied k-means clustering algorithm based on the *ETs*. When we used *total* energy as a single feature, the accuracy of clustering for class A was no more than 0.52, while the accuracy for class B was much more, i.e. 0.76. For classes C and D, which include both types of secondary structures, the accuracies were between those of A and B. Using all *ETs* we obtained similar results. Therefore, we decided to unify the method for all structural classes. We combined two most differentiating *ETs* from each class and considered them as common indicators for all classes. As a results, the k-means clustering algorithm used three common *ETs*: probability of amino acid at *Φ* and *Ψ*, Ramachandran preferences and Coulomb interactions. The accuracies of clustering with these energy terms were from 0.68 for class A to 0.76 for class C, with sensitivity and selectivity in the range from 0.68 to 0.87.

A great advantage of our approach is using the same methodology to all classes of protein structures. Clustering based on the common *ETs* does not even require the knowledge about the secondary structure. The models may be ranked in two clusters separately, allowing to choose the best natively oriented and mirror models for further analysis. The method can be applied to all fully-automated tools for protein structure reconstruction based on contact maps, especially those analyzing big sets of models.

## Materials and methods

### Data set

The data set was built from representatives of SCOP [19] superfamilies. 1961 domains were downloaded from on-line SCOP server (http://astral.berkeley.edu/scopseq-1.75.html as of 20.09.2012). The domains with special proteinogenic amino acids, such as selenocysteine, in the middle of the chain were eliminated. Some domains had missing residues or heavy atoms in the middle of the chain. They were also rejected from the further analysis. If the special amino acids were at the beginning or end of the chain, the domain was modified by the reduction of the chain. The same procedure was applied in the case of absence of heavy atoms at the beginning or end of the chain. Finally, 1305 domains from 7 classes, which constitutes 67% of the data set, were used in the experiment (Table 3).

**Table 3:** Descriptions of data set.

SCOP class	Class description	Domains for structural analyses	Domains for energy analyses: at least 3 mirror and 3 native models	Mean sequence length ± std
A	*All-alpha*	343	329	124 ± 86
B	*All-beta*	233	218	153 ± 98
C	*Alpha/beta* (mainly parallel beta sheets—beta-alpha-beta units)	149	140	228 ± 105
D	*Alpha+beta* (mainly antiparallel beta sheets—segregated alpha and beta regions)	368	352	132 ± 74
E	*Multi-domain* (alpha and beta—folds consisting of two or more domains belonging to different classes)	21	15	354 ± 183
F	*Membrane and cell surface proteins and peptides* (does not include proteins in the immune system)	78	74	137 ± 126
G	*Small proteins* (usually dominated by metal ligand, heme, and/or disulfide bridges)	113	113	57 ± 24
**All**		**1305**	**1241**	**142± 101**

To build the data set of models with two orientations and various qualities, we used the procedure of modeling a structure from its contact map. For each domain the contact map was generated with PconPy [20] with the following parameters: the cutoff of distance between Cα atoms was 8 Å, and separation between residues was 1. Next, the contact maps were used as an input for C2Sv2.0 [10,8,21,22] to reconstruct structural models. C2Sv2.0 uses REMO [21] to backbone reconstruction. Therefore the models are obtained using knowledge-based potentials deduced from solved protein structure, which are oriented correctly, hence the models are not ideal mirror reflections of natively oriented domains.

For each of the selected domains 100 models were generated.

### Structural features

For structural assessment of the models the root mean square deviations (RMSDs) between Cα atoms were calculated. The RMSD is sensitive to global changes in the structure so it should be effective to assess the symmetric differences, such as mirror images. The structural correctness was also evaluated with the number of positive dihedral angles *Φ*. The *Φ*^*+*^ *ratio* was calculated as a ratio of the number of positive dihedral angles, *Φ*^*+*^, to all dihedral angles, *Φ*. All calculations were made with Biopython [23,24].

The models could have two orientations: native or mirror. The orientation of a model was assessed based on its superposition to its original SCOP structure and to the mirror image of the SCOP structure. The mirror image of the SCOP structure was obtained by geometric, symmetric reflection. We used the superposition algorithm from Biopython. Only Cα atoms were superposed and RMSDs between Cα atoms were calculated. The orientation class of the model was assigned depending on the lower RMSD value of these two superpositions.

The Ramachandran plots of the models were made with *ramachandran* function [25] of Matlab R2013a Bioinformatics toolbox. Calculations of the residues occupancy in different regions of Ramachandran plots were carried out based on the *Top500 angle data* [26]. *Top500 angle data* is a selection of 500 files from the Protein Data Bank prepared by the Richardson lab from Duke University [27]. The list of proteins is available at the website: http://kinemage.biochem.duke.edu/databases/top500.php. The classification of regions was made based on the Richardson lab guidelines. Density levels were classified by the fraction of data points excluded. The favored, allowed and outliers regions were defined with the minimum occupancy grid values (*p*_*ΦΨ*_):

$$favored=p_{\phi \psi }\geq 0.02$$ (1)

$$allowed={p_{\phi \psi }<0.02\wedge p}_{\phi \psi }\geq 0.0005$$ (2)

$$outliers=p_{\phi \psi }<0.0005$$ (3)

In structural analysis of the models we tested if contact maps of the models remained the same as the original contact maps from which the models were reconstructed. For each model the contact map was generated with PconPy with the following parameters: cutoff distance between Cα atoms was 8 Å, and separation between residues was 1. They included the matrix representation of contact maps, where 1 means contact and 0 means non-contact. All normalizations were made comparing to the original SCOP domains. The normalized differences (*diff*) in the contact number between a model and its original SCOP domain were calculated.

### Energy terms

After investigation of the structural differences between native and mirror models, we verified if methods based on the *ETs* may be suitable for distinguishing between two orientations of the models. PyRosetta package [28] was selected to calculate the energy stability of a protein structure. We used *talaris2013* energy score function [29], where the total energy of a model is the weighted sum of 16 *ETs*. The *ETs*, along with their short descriptions, are listed in Table 4. Some energy terms describe empirical potentials (e.g. Coulomb interaction, Lennard-Jones potentials) and some of them are knowledge-based potentials (e.g. Ramachandran preferences, probability of an amino acid at *Φ* and *Ψ*).

**Table 4:** Description of the energy terms from PyRosetta used in the analysis.

ET shortcut	ET description
*fa_atr*	Lennard-Jones attractive
*fa_rep*	Lennard-Jones repulsive
*fa_sol*	Lazardis-Karplus solvation energy
*fa_intra_rep*	Lennard-Jones repulsive between atoms in the same residue
*hack_elec*	Coulomb interaction
*pro_close*	proline ring closure energy
*hbond_sr_bb*	backbone-backbone hydrogen bonds close in primary sequence
*hbond_lr_bb*	backbone-backbone hydrogen bonds distant in primary sequence
*hbond_bb_sc*	sidechain-backbone hydrogen bond energy
*hbond_sc*	sidechain-sidechain hydrogen bond energy
*dslf_fa13*	disulphide bonds energy
*rama*	Ramachandran preferences
*omega*	omega dihedral in the backbone
*fa_dun*	internal energy of sidechain rotamers as derived from Dunbrack's statistics
*p_aa_p*	probability of amino acid at Φ and Ψ
*ref*	reference energy for each amino acid
*total*	final score (total energy)

### Assessment of energy terms usability

**Fig 7:**
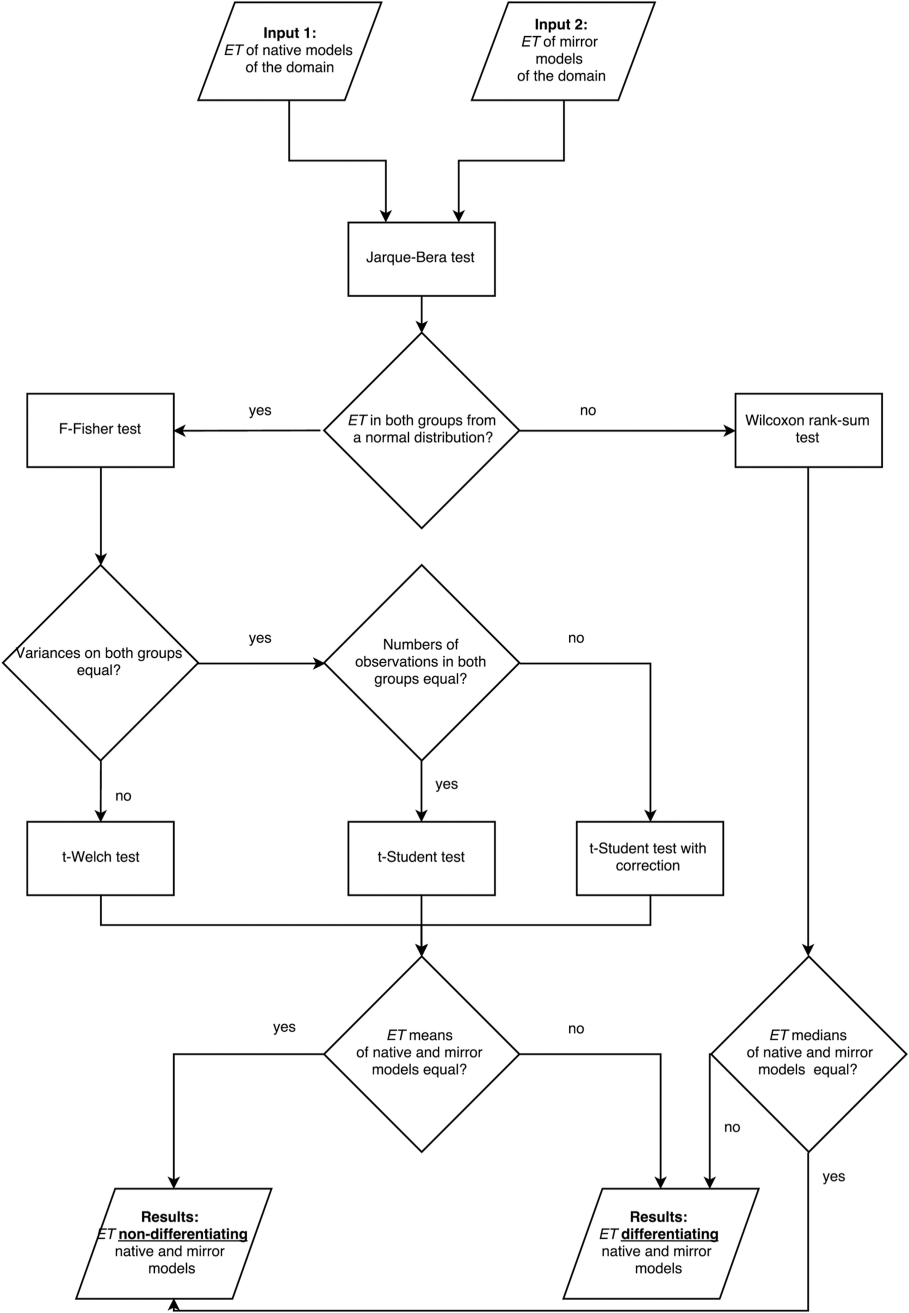
Statistical analyses of the energy terms in the groups of natively oriented and mirror models. The finally tested hypothesis was if the energy term of native and mirror models were the same.

The basic hypothesis to be verified was whether a certain *ET* is significantly different in natively oriented and mirror models of a protein domain. The statistical analyses were performed with Matlab R2013a. The schema of the statistical analysis of *ETs* between native and mirror models is shown in Fig 7. Each analysis was preformed individually for each domain. Next, the ratio of domains, for which the differences between natively oriented and mirror models were statistically significant, was calculated for each *ET*. Furthermore, *Φ*^*+*^ *ratio* was included to the *ETs* analysis in the same way.

After analysis of statistical differences between *ET* values of native and mirror models, the relation between *ET*s was calculated:

$${RET}_{\frac{n}{m}}=\frac{\bar{ETn}}{\bar{ETm}}$$ (4)

$\bar{ETn}$ – mean or median of an *ET* of native models of a domain,

$\bar{ETm}$ – mean or median of an *ET* of mirror models of a domain.

### Clustering models based on energy terms

We proposed an automatic method to distinguish between native and mirror models. For this purpose we clustered models based on their ETs. In the first part of the study we tested which energy terms are significantly different for native and mirror models. Using statistical tests we compared the medians or means of native models of one domain and mirror models of the same domain. Next, we repeated this procedure for all domains from each class and calculated the ratio of domains in which the energy term was significantly different. In the second part of the study, we used the energy terms as features in k-means clustering. We clustered 100 models from each domain and compared results with the actual model orientation. Then, we calculated accuracy, specificity, and sensitivity. We repeated procedure for all domains from each class, and calculated the mean of accuracy, specificity, and sensitivity.

Clustering was performed with *kmeans* function of Matlab R2013a. The data set of models of each domain was divided into 2 clusters with *sqeuclidean* measure. First, for the clustering we used all *ETs* as features of all the structural models. Next, we carried out a series of analyses with selected *ETs*, dividing the data set into two clusters. The cluster with a lower mean value of tested *ETs* was assumed as the native cluster, and the other one as the mirror cluster. To assess the clustering results, the accuracy (ACC), specificity (SPC), sensitivity (SN), Matthews correlation coefficient (MCC) and F1 score (F1) were calculated:

$$ACC=\frac{TP+TN}{TP+TN+FP+FN}$$ (5)

$$SPC=\frac{TP}{TP+FN}$$ (6)

$$SN=\frac{TN}{TN+FP}$$ (7)

$$MCC=\frac{TP∙TN-FP∙FN}{\sqrt{\left(TP+FP)(TP+FN)(TN+FP)(TN+FN\right)}}$$ (8)

$$F1=2∙\frac{1}{\frac{1}{SN}+\frac{TP+FP}{TP}}$$ (9)

where *TP* is a number of true positives, which are models with the native orientations of their structures assigned to the cluster classified as native; *TN* is a number of true negatives, which are mirror models in the mirror cluster; *FP* is a number of false positives, which are mirror models in the native cluster; *FN* is a number of false negatives which are native models in the mirror cluster.

The parameters can assume values between 0 and 1, where 1 denotes the ideal clustering case in which all structural models are in the right cluster. ACC, SPC, SN, MCC and F1 were calculated for each domain and finally averaged over all domains.

## Supporting information

S1 Fig Scatterplot of mean RMSD of native models to the original SCOP structure (green *) and mean RMSD of mirror models to the mirror SCOP image (red *) for each domain from A class. (TIFF)

S2 Fig Bar graph showing the ratio of domains from E, F and G classes for which the ET was significantly different in the groups of natively oriented and mirror models. Graph includes also *Φ*^*+*^ *ratio*.
(TIFF)

S3 Fig The histograms of RET for the three best ETs in distinguishing native and mirror models. Left column is class C and right column is class D, red bars mean the domain for which the ET was significantly different and blue bars mean the domain for which the ET was not significantly different.
(TIFF)

S4 Fig The histograms of RET for the four best ETs in distinguishing native and mirror models in class E. (TIFF)

S5 Fig The histogram of RET for the best ET in distinguishing native and mirror models in class G. (TIFF)

S1 Table Models with unchanged contact maps compared to the original SCOP structure. (PDF)

S2 Table Mean accuracy of the k-means clustering using energy terms for classes E, F and G. (PDF)
