# Supplementary figures and images for: Automated method to differentiate between native and mirror protein models obtained from contact maps
Source: PLoS One. 2018 May 22;13(5):e0196993. doi: 10.1371/journal.pone.0196993 (PMC5963800; doi:10.1371/journal.pone.0196993)

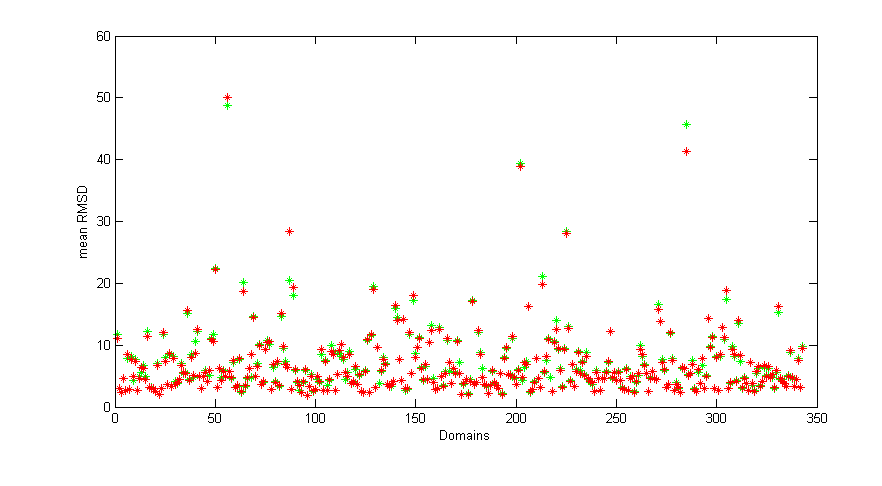

Supplement: S1 Fig — (TIFF) [file pone.0196993.s001.tiff]

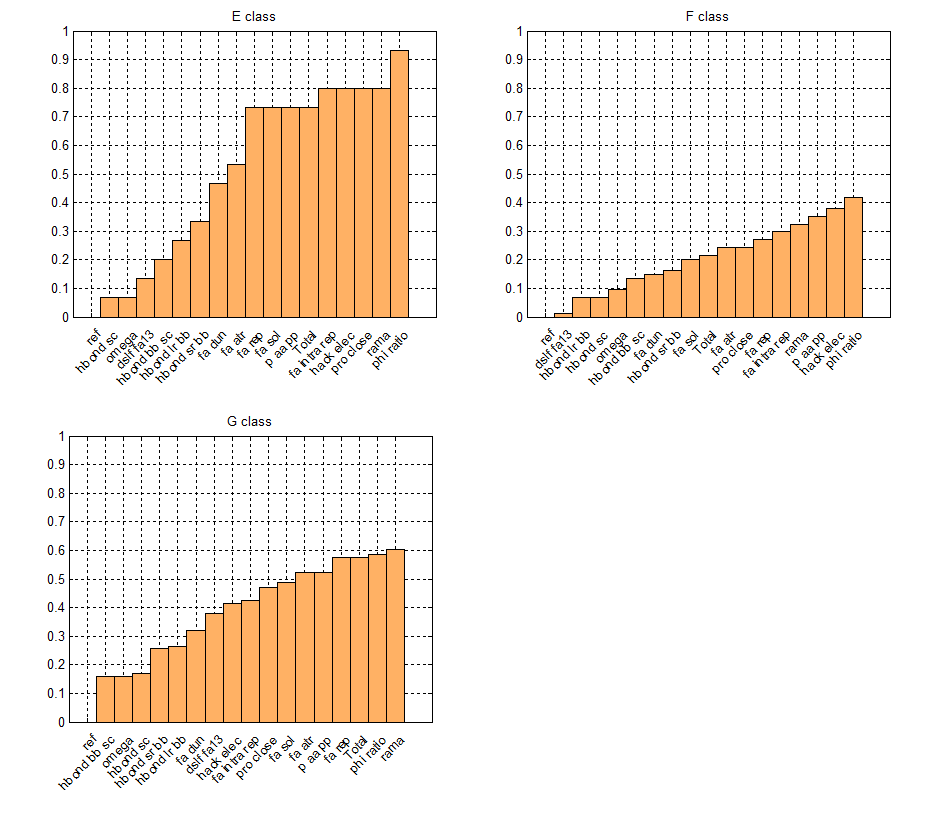

Supplement: S2 Fig — Graph includes also Φ+ ratio. (TIFF) [file pone.0196993.s002.tiff]

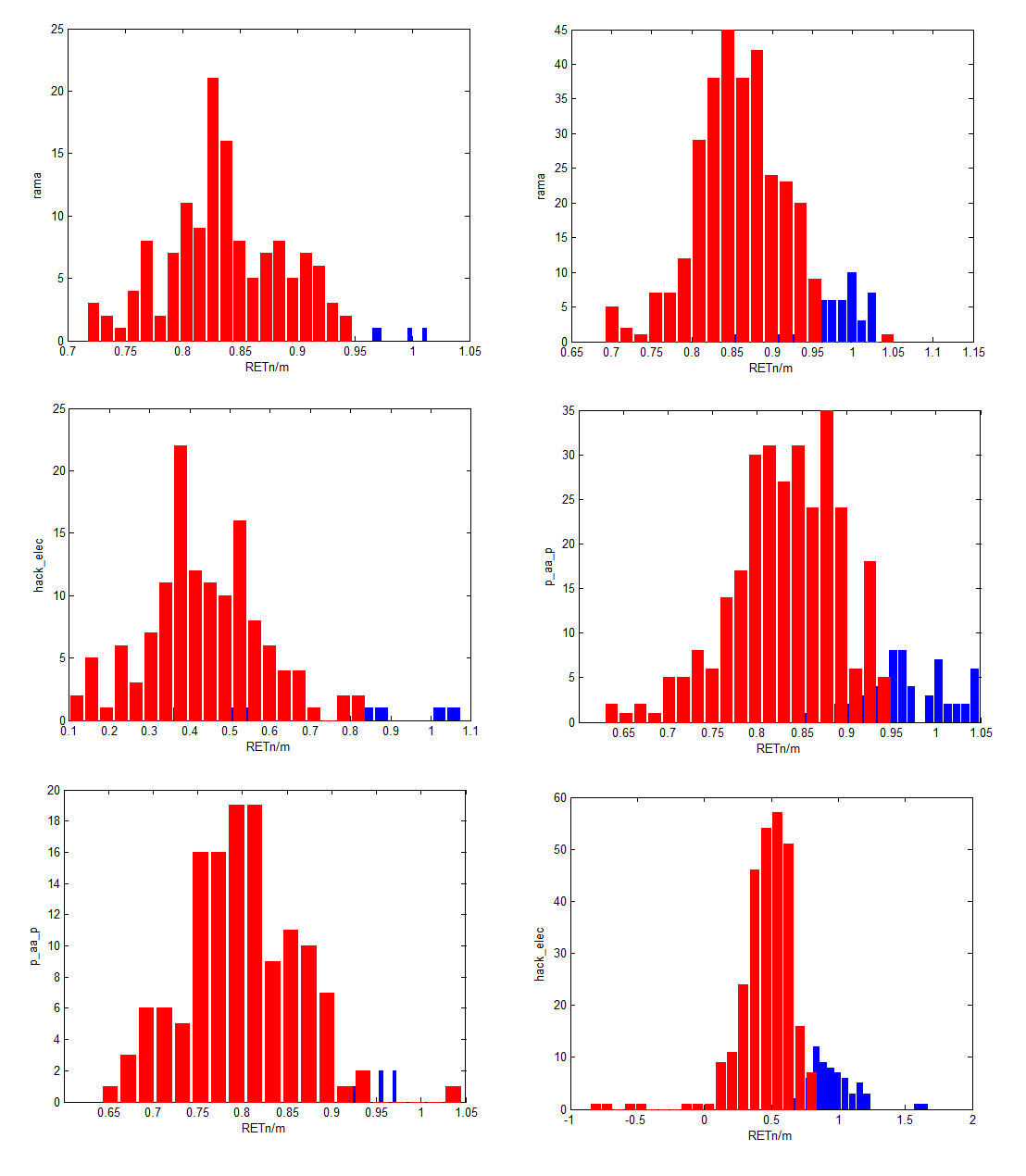

Supplement: S3 Fig — Left column is class C and right column is class D, red bars mean the domain for which the ET was significantly different and blue bars mean the domain for which the ET was not significantly different. (TIFF) [file pone.0196993.s003.tiff]

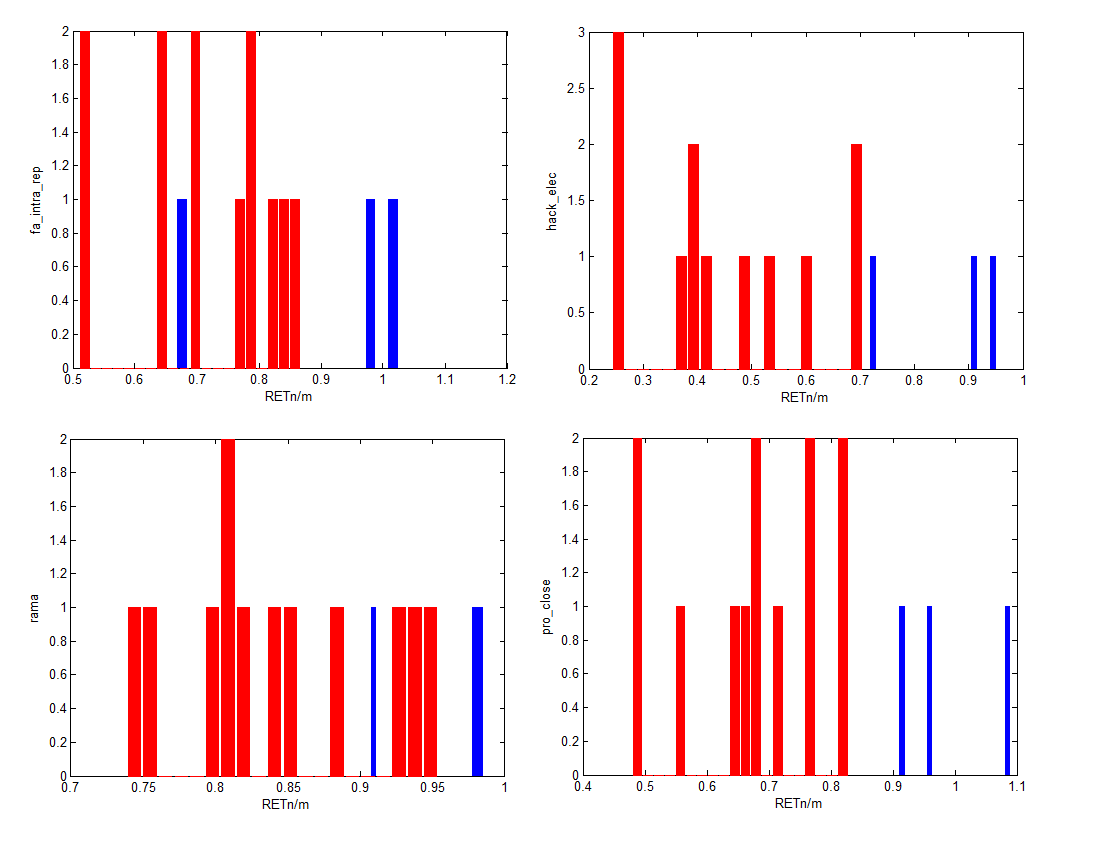

Supplement: S4 Fig — (TIFF) [file pone.0196993.s004.tiff]

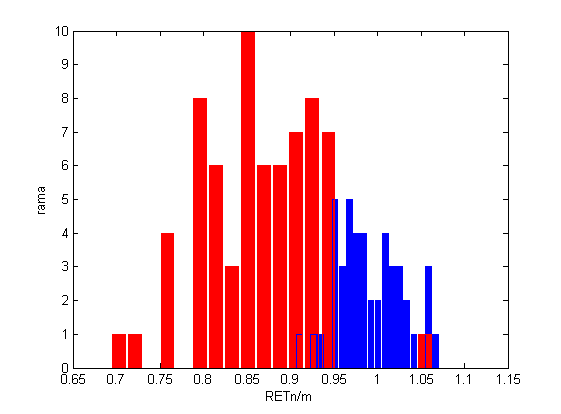

Supplement: S5 Fig — (TIFF) [file pone.0196993.s005.tiff]
