# Supplementary material for: Automated method to differentiate between native and mirror protein models obtained from contact maps
Source: PLoS One. 2018 May 22;13(5):e0196993. doi: 10.1371/journal.pone.0196993 (PMC5963800; doi:10.1371/journal.pone.0196993)
Supplement: S1 Table — (PDF) [file pone.0196993.s006.pdf]

Supplementary Table 1. The portion of models with unchanged contact maps compared to the original SCOP structure.

SCOP classes	Unchanged	Changed < 1%	Changed < 5%
A	5%	54%	78%
B	16%	82%	93%
C	2%	71%	89%
D	9%	73%	89%
E	1%	56%	70%
F	21%	61%	82%
G	17%	56%	88%
**All**	**10**%	**67**%	**86**%
