# Supplementary material for: Automated method to differentiate between native and mirror protein models obtained from contact maps
Source: PLoS One. 2018 May 22;13(5):e0196993. doi: 10.1371/journal.pone.0196993 (PMC5963800; doi:10.1371/journal.pone.0196993)
Supplement: S2 Table — (PDF) [file pone.0196993.s007.pdf]

*Supplementary Table 2. Mean accuracy of the k-means clustering using energy terms for classes E, F and G.*

| SCOP class | | E | F | G |
|---|---|---|---|---|
| All *ETs* | *ACC* | 0.65 | 0.54 | 0.61 |
| | *SPC* | 0.66 | 0.49 | 0.55 |
| | *SN* | 0.71 | 0.60 | 0.59 |
| | *MCC* | 0.32 | 0.08 | 0.23 |
| | *F1* | 0.66 | 0.56 | 0.61 |
| *ETs* different for > 60% domains | *ACC* | 0.65 | - | 0.61 |
| | *SPC* | 0.65 | - | 0.63 |
| | *SN* | 0.70 | - | 0.61 |
| | *MCC* | 0.30 | - | 0.23 |
| | *F1* | 0.63 | - | 0.59 |
| 2 most differentiating *ETs* | *ACC* | 0.68* | 0.60 | 0.60 |
| | *SPC* | 0.76* | 0.59 | 0.53 |
| | *SN* | 0.71* | 0.62 | 0.70 |
| | *MCC* | 0.34* | 0.19 | 0.23 |
| | *F1* | 0.65* | 0.60 | 0.62 |
| 1 most differentiating *ETs* | *ACC* | 0.68* | 0.59 | 0.61 |
| | *SPC* | 0.76* | 0.56 | 0.63 |
| | *SN* | 0.71* | 0.64 | 0.61 |
| | *MCC* | 0.34* | 0.34 | 0.22 |
| | *F1* | 0.65* | 0.64 | 0.59 |
| 1 *ET*: *total* | *ACC* | 0.66 | 0.54 | 0.61 |
| | *SPC* | 0.65 | 0.44 | 0.53 |
| | *SN* | 0.71 | 0.62 | 0.71 |
| | *MCC* | 0.30 | 0.30 | 0.23 |
| | *F1* | 0.63 | 0.63 | 0.63 |
| 3 *ETs*: *hack_elec, p_aa_p, rama* | *ACC* | 0.70 | 0.59 | 0.60 |
| | *SPC* | 0.84 | 0.59 | 0.62 |
| | *SN* | 0.68 | 0.60 | 0.61 |
| | *MCC* | 0.44 | 0.19 | 0.22 |
| | *F1* | 0.68 | 0.59 | 0.59 |

The models were divided into 2 classes. Only domains with at least 3 mirror and 3 native models were included. Squared Euclidean distance was used. ACC denotes accuracy (Eq. 5), SPC denotes specificity (Eq. 6), SN denotes sensitivity (Eq. 7), MCC denotes Matthews correlation coefficient (Eq. 8), and F1 denotes F1 score (Eq. 9).

* The results of clustering in class E for 2 most differentiating *ETs* and 1 most differentiating *ETs* are the same, because the 4 *ETs* had the same values of the domains for which were significantly different. For that reason they were taken all to cluster as 2 most differentiating *ETs* and 1 most differentiating *ETs*.
